# Sparse ECG Denoising with Generalized Minimax Concave Penalty

**DOI:** 10.3390/s19071718

**Published:** 2019-04-10

**Authors:** Zhongyi Jin, Anming Dong, Minglei Shu, Yinglong Wang

**Affiliations:** 1 Shandong Provincial Key Laboratory of Computer Networks, Shandong Computer Science Center (National Supercomputer Center in Jinan), Qilu University of Technology (Shandong Academy of Sciences), Jinan 250014, China; zyjin.qlut@gmail.com (Z.J.); wangylscsc@126.com (Y.W.); 2 School of Computer Science and Technology, Qilu University of Technology (Shandong Academy of Sciences), Jinan 250353, China

**Keywords:** ECG denoising, sparse recovery, Generalized Minimax Concave Penalty (GMC), *ℓ*_1_-norm

## Abstract

The electrocardiogram (ECG) is an important diagnostic tool for cardiovascular diseases. However, ECG signals are susceptible to noise, which may degenerate waveform and cause misdiagnosis. In this paper, the ECG noise reduction techniques based on sparse recovery are investigated. A novel sparse ECG denoising framework combining low-pass filtering and sparsity recovery is proposed. Two sparsity recovery algorithms are developed based on the traditional $\ell_{1}$-norm penalty and the novel generalized minimax concave (GMC) penalty, respectively. Compared with the $\ell_{1}$-norm penalty, the non-differentiable non-convex GMC penalty has the potential to strongly promote sparsity while maintaining the convexity of the cost function. Moreover, the GMC punishes large values less severely than $\ell_{1}$-norm, which is utilized to overcome the drawback of underestimating the high-amplitude components for the $\ell_{1}$-norm penalty. The proposed methods are evaluated on ECG signals from the MIT-BIH Arrhythmia database. The results show that underestimating problem is overcome by the proposed GMC-based method. The GMC-based method shows significant improvement with respect to the average of output signal-to-noise ratio improvement ($SNR_{imp}$), the average of root mean square error (RMSE) and the percent root mean square difference (PRD) over almost any given SNR compared with the classical methods, thus providing promising approaches for ECG denoising.

## 1. Introduction

As the recording of the bioelectric potentials produced by rhythmical cardiac activities, the Electrocardiogram (ECG) signals provides plentiful and valuable information on the heart functional conditions, and it is thus has been widely used not only for the clinical diagnosis of cardiovascular diseases in hospital, but also for patient monitoring at home. However, the ECG signals are essentially weak and non-stationary, and so susceptible to various noises. The noise may blur or even mask the key characteristics in the ECG waveform, which will further degenerate the reliability and efficiency of the clinical diagnosis. Therefore, it is important and valuable to denoise the ECG, i.e., extract high resolution cardiac signals from noisy ECG observations.

ECG noise elimination is a very challenging task due to the spectral overlap of signals and noise, and a wide range of ECG denoising techniques have been continuously and extensively studied in the last few decades. These methods can be typically classified as the linear filtering, empirical mode decomposition (EMD) and wavelet transform, etc. They are the most well-developed approaches and have their own advantages and drawbacks.

The linear filtering methods [1,2,3,4] have low computational complexity and fast calculation speed and thus have been widely used in embedded or portable monitoring instruments. However, the classical filters failed to reduce the noise components that overlap with signal spectrum. Moreover, they will cause significant damage to the details of ECG signals which contain sharp edges and impulses of short duration. Therefore, they are often used in combination with other noise reduction algorithms.

To overcome these difficulties, nonlinear methods have been investigated and Wavelet-based methods are the most famous and effective [5,6,7,8,9,10,11,12,13,14]. These approaches rely on the idea of thresholding under the assumption that signal magnitudes will concentrate in the Wavelet representation while the noise will spread, such that the noise can be illuminated by cutting the Wavelets coefficients less than a pre-determined threshold. The performance of the Wavelet denoising methods depends on the matching degree of the selected Wavelet basis functions and the ECG signals. The basis functions will be fixed once the algorithms have been developed and may not necessarily match the practical ECG signals.

Due to the ability of dealing with non-stationary and nonlinear signals, the EMD-based ECG denoising methods gained extensive attention recently [15,16,17,18,19,20,21]. The EMD method was first introduced in [22] for analyzing data from nonstationary and nonlinear processes. The EMD decomposes a given signal into a series of intrinsic mode functions (IMFs) through an iterative process called sifting. The IMFs play the roles of basis functions, and the EMD method can be seen as a type of Wavelet decomposition with adaptive basis functions. Since the basis functions of the EMD are derived from the signal itself, it has the potential to outperform the traditional Wavelet methods. However, the EMD has a limitation of mode mixing, which causes the mixing of higher order components with the lower order components. Because of the mode mixing problem, it is difficult to separate the signal and noise components clearly even in higher order IMFs. To overcome this problem, paper [21] proposed a grey spectral noise cancellation (GSNC) scheme for ECG signals where two-stage discrimination is employed with the empirical mode decomposition (EMD), the ensemble empirical mode decomposition (EEMD) and the grey spectral noise estimation (GSNE). This method effectively alleviates the mode mixing problem. In the case of denoising, removing some of the IMFs will inevitably corrupt the useful information of the signal [23]. For example, in ECG denoising, the EMD may cause damage to P-waves and T-waves, which may result in misdiagnosis [24].

Either the Wavelet or the EMD methods can be classified as the transform-domain processing approaches under the assumption that the true signal can be well approximated by a linear combination of some basis functions [25,26]. The essence underlying these methods is that the signal can be sparsely represented in the transform domain. Since the ECG signals consist of periodical pulse components, they can also be well represented by a combination of low pass components and sparse components in time domain. Consequently, ECG denoising methods based on the time-domain sparse representations have been an active research area in recent years. Paper [27] modeled the ECG signals as the sum of two second and third order sparse derivative signals to reduce noise. In [28], an sparse ECG signals enhancement and QRS waves detection algorithm was proposed. In [29,30], the sparse optimizationis combined with linear time invariant (LTI) filtering to conduct ECG signals denoising. In these sparse representation methods, $\ell_{1}$-norm is used as a penalty term to construct sparse optimization models. This penalty term has the ability to control the sparsity of the optimization results. However, the $\ell_{1}$-norm penalty has a drawback that it always underestimates the interested signal [31], since larger coefficients are penalized more heavily in the $\ell_{1}$ norm than smaller coefficients [32]. The underestimating causes the amplitude of estimated signals to be lower than the true value, which may lead to misdiagnoses.

In order to improve the denoising performance, a variety of non-convex penalty terms have been developed to replace $\ell_{1}$-norm [32,33,34]. Paper [32] proposes a weighted formulation of $\ell_{1}$-norm minimization designed to more closer to the solution of $\ell_{0}$-norm. In [33], the author uses $\ell_{2}-\ell_{0}$ penalty instead of $\ell_{1}$-norm penalty. Paper [34] addresses the minimization problem of $\ell_{0}$-regularized least squares for a continuum and propose two heuristic search algorithms. However, these methods adopt non-convex penalty to replace $\ell_{1}$-norm, which leads to nonconvex objective functions. As a result, the algorithms become complicated and the solution is often stuck in the local optimal solutions. To overcome these drawbacks, a novel multivariate non-separable non-convex penalty, namely generalized minimax-concave (GMC) penalty, has been recently presented in [35,36]. The GMC penalty is a non-differentiable non-convex penalty obtained by subtracting a differentiable generalized Huber function from $\ell_{1}$-norm. It strongly promotes sparsity while maintaining the convexity of the cost function.

Currently, the GMC penalty has been successfully applied to the fault detection of gearbox and achieved good results [31,37]. However, the application of GMC penalty to the ECG denoising has not been well studied yet. It is not known whether the GMC penalty can be applied to the ECG signals processing area to improve the denoising performance. This motivates the work in this paper to explore the GMC-based sparse denoising method.

In this paper, we study the sparse denoising techniques for the ECG signals. Through modeling the observed ECG signals as a sum of low-pass component, sparse component and additive noise component, we propose an ECG denoising framework by combining the LTI low-pass filtering and the sparsity recovering. As the key ingredients of the framework, the sparsity recovery algorithms are investigated and we proposed two sparsity recovery algorithms based on $\ell_{1}$-norm penalty and GMC penalty, respectively. We compare the denoising performance of the proposed two schemes and demonstrate that the GMC-based scheme preservers more detail information with respect to QRS waves of ECG signals. The contributions of this work are summarized as follows.

1. A novel ECG denoising framework based on sparsity recovery is proposed. Low-pass filtering and sparse denoising idea was first proposed in [38], but it is not known if it can be applied to the ECG denoising. Moreover, the sparsity in [38] deals with the derivatives of the signal, while we directly deal with the sparsity components of the signal. Therefore, we propose a different sparse framework for ECG denoising.
2. We extend the application of GMC penalty to the area of ECG denoising. Although the GMC penalty has been proposed in [35] and several application examples were given there, it is not known how to apply it in the ECG area and whether the performance of ECG denoising can be improved.
3. The performance of the GMC-based sparse denoising algorithm is comprehensively tested. We carry out a series of experiments to explore the performance the GMC-based denoising algorithm. The relationship between the parameter setting and the performance are demonstrated through experiments. It is also shown that the GMC-based algorithm helps to overcome the underestimation problem and outperforms the classical methods known in the literature.

The paper is organized as follows. We introduce the sparsity recovery denoising framework in Section 2. The $\ell_{1}$-norm based algorithm is described in Section 3. The GMC-based sparse denoising algorithm is proposed in Section 4. Experimental results are presented in Section 5. Section 6 concludes the work.

## 2. System Model

In this section, the sparse signal model in time domain is presented. A sparse denoising framework based on this signal model is established with the help of mathematical derivations.

### 2.1. Signal Model

The noisy ECG signals can be modeled as the superposition of a low-pass signal, a sparse signal and the additive white Gaussian noise (AWGN), which can be written as

(1) $$\begin{array}{c} y(n)=l(n)+d(n)+w(n),\;\;\;\;n\in Z \end{array}$$

where *n* is the index to the discrete sampling sequences, l(n), d(n) and w(n) represent the low-pass signal, the sparse signal and the additive white Gaussian noise (AWGN), respectively. The ’sparse’ property means that there are many zeros in a signal frame with *N* data points. To easily understand, we can regard d(n),0,1,2,….,N−1, as the periodic components in the ECG signal frame. In the following, to facilitate our derivations, we omit the time index (n) in the case of not being confused.

### 2.2. Denoising Framework

We aim to recover the pure ECG signals l+d from the observed noisy signals *s*. To achieve this, a straightforward method is the traditional linear filtering procedure. Specifically, let s≜l+d represent the pure ECG signals, then it can be approximately estimated through traditional linear filtering

(2a)LPF(y)=LPF(l+d+w),(2b)=LPF(l+w)+LPF(d),(2c)=l´+LPF(d),

where LPF represents a low-pass filtering (LPF) process and $\overset{´}{l}≜LPF(l+w)$. Here, we use the zero-phase filter since it can reduce noise in the signals and preserves the QRS complex at the same time, it occurs in the original [38,39]. To facilitate readers, the design method of the LPF is sketched in Appendix A.

From Equation (2c), it is observed that the traditional LPF will smooth not only the low-pass component but also the sparse component in the original signals. Since the sparse component *d* consists of high frequency ingredients, it is prone to be damaged by the LPF, which will be embodied by the injury of the details of the ECG signals. For example, the amplitude of the peaks, the shape of the Q and R waves may be damaged by the LPF.

**Figure 1:**
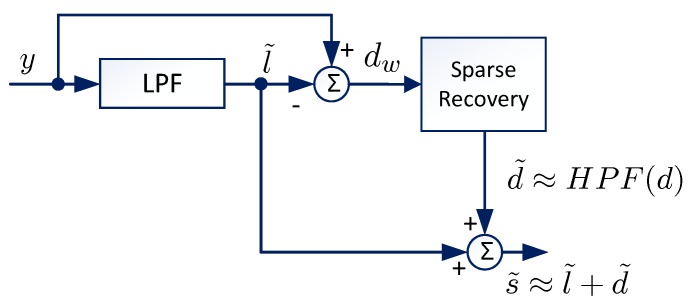
Block diagram of the sparsity electrocardiogram (ECG) denoising process.

As mentioned above, the traditional LPF methods may cause damage to the details of the ECG signals due to the high-frequency ingredients. In order to enhance the details in the recovered signals, we propose a new ECG denoising framework as shown by Figure 1. The main idea is to recover these details, which is sparse, from the residual of the low-pass filter. Specifically, the residual of the LPF is written as

(3a)y−LPF(y)=HPF(y),(3b)=HPF(l+d+w),(3c)=HPF(d)+HPF(l+w),(3d)=HPF(d)+w˜,

where HPF represents a high-pass filter (HPF). It is assumed that the LPF and HPF are compensative, i.e., HPF=I−LPF. Here, *I* represents the identity operator. From Equation (3d), we know that the residual of the LPF consists of the high-passed ingredient of the sparse signals plus high-frequency noise. If $\tilde{d}≜HPF\left(d\right)$ is recovered, then it can be combined with the output of the LPF ( $\tilde{l}=\overset{´}{l}+LPF\left(d\right)$) as shown in Figure 1. The combined signal can be written as

(4a)s˜=l˜+d˜,(4b)≈l´+LPF(d)+HPF(d).

Recall that HPF(d) contains the details of the sparse high-frequency components, the details of the recovered ECG signals can be thus improved. Then the problem boils down to how to recover this high-frequency sparse signals, i.e., HPF(d), from noise.

In the following, we propose two methods to recover the sparse components in the residual of the LPF. The first one is based on the $\ell_{1}$-norm penalty, which improves the details in the detains of the output of LPF but still has obvious underestimating phenomenon. The second one is based on the GMC penalty, which achieves better performance than the $\ell_{1}$-norm method and overcomes the underestimating problem.

## 3. Sparsity Recovery with $\ell_{1}$-Norm

In this section, we propose the $\ell_{1}$-norm based sparsity recovery algorithm for the sparsity recovery block in Figure 1.

From Figure 1, we know that the performance of the sparsity recovery plays the vital role in the whole denoising procedure. The classical sparsity recovery methods can be applied here straightforwardly, e.g., the $\ell_{1}$-norm penalized least squares method [40]. Specifically, the sparse signal $\tilde{d}$ can be decomposed as

(5) $$\begin{array}{c} \tilde{d}=Ax\;, \end{array}$$

where $A\in R^{N\times M}$ is the basis vectors of the signal space and $x\in R^{M}$ is the representation coefficients. Given *A*, we expect to find a sparse solution *x* to satisfy the equality. The sparse solving process can be mathematically reformulated as the following $\ell_{1}$-norm optimization problem

(6) $$\begin{array}{cc} \min_{x} & {\;∥x∥}_{1}\\ s.t.: & \;\tilde{d}=Ax\;. \end{array}$$

Note that *A* can be constructed using different methods in principle [35]. In this work, we construct *A* based on the Short Time Fourier Transform (STFT) matrix as shown in Appendix B, considering that the ECG signals are periodic pulses in time series.

When $\tilde{d}$ is corrupted by noise, i.e., $d_{w}≜\tilde{d}+\tilde{w}$ is observed, an approximation solution to Equation (6) can be found by solving the following basis pursuit denoising (BPD) problem [41,42]

(7) $$\begin{array}{c} \arg\min_{x}\frac{1}{2}∥d_{w}{-Ax∥}_{2}^{2}+\lambda {∥x∥}_{1}\;. \end{array}$$

Specifically, the forward-backward spliting (FBS) method can be applied to find the solution of Equation (7) [43], as listed in Algorithm 1. It is noted that the soft(·) in the algorithm is the soft thresholding operator defined by

(8) $$\begin{array}{c} soft(t,T)=t*max\{0,1-T/|t|\}\;, \end{array}$$

where $T\in R_{+}$ is a given threshold. The maxeig(·) denotes the maximum eigenvalue of a matrix.

Our simulation results show that the details in the recovered ECG signals of the proposed $\ell_{1}$-norm method are enhanced significantly compared with the output of LPF. However, there still exists an obvious gap between the recovered signal and the noiseless signal with respect to the peaks. That is to say the proposed $\ell_{1}$-norm method underestimates the peaks values in the signals. This drawback of the $\ell_{1}$-norm methods has also been revealed in literature [31,35]. To further improve the performance of the denoised signals with respect to the details, we propose a non-convex sparse regularization method based on the generalized minimax concave (GMC) penalty in the following.

**Algorithm** **1** FBS algorithm for $\ell_{1}$-norm penalized least squares problem**Input:** $x^{\left(0\right)},A,A^{T},\lambda$**Output:** $x^{\left(i+1\right)}$
1:**Set** $\rho =maxeig(∥A^{T}A∥)$2:**Set** μ:0<μ<1/ρ3:Number of iteration: $N_{iter}$4:**for** $i=0\;to\;N_{iter}$**do**5: $w^{\left(i\right)}=x^{\left(i\right)}-\mu A^{T}\left(Ax^{\left(i\right)}-d_{w}\right)$
6: $x^{\left(i+1\right)}=soft\left(w^{\left(i\right)},\mu \lambda \right)$
7:
**end for**
8:**return** $x^{\left(i+1\right)}$


## 4. Sparsity Recovery with GMC Penaly

As mentioned above, the classical sparsity recovery method based on $\ell_{1}$-norm penalty will lead to underestimation to the peaks values. In this section, instead of the $\ell_{1}$-norm penalty, we adopt a novel penalty for the sparsity recovery problem, which can keep the convexity of the least squares cost function minimized and avoids underestimation the values of waveform.

### 4.1. GMC Penalty

The GMC penalty function $\psi_{B}:R^{N}\to R$ is defined as

(9) $$\begin{array}{cc} \psi_{B}\left(x\right) & ={∥x∥}_{1}-S_{B}\left(x\right)\\ & ={∥x∥}_{1}-\min_{v\in R^{N}}{\{∥v∥}_{1}+\frac{1}{2}{∥B\left(x-v\right)∥}_{2}^{2}\}\;, \end{array}$$

where, $B\in R^{M\times N}$ and $S_{B}≜\inf_{v\in R^{N}}{\{∥v∥}_{1}+\frac{1}{2}{∥B\left(x-v\right)∥}_{2}^{2}\}$ is the generalized Huber function [35], inf{·} denotes the infimum of a function.

**Figure 2:**
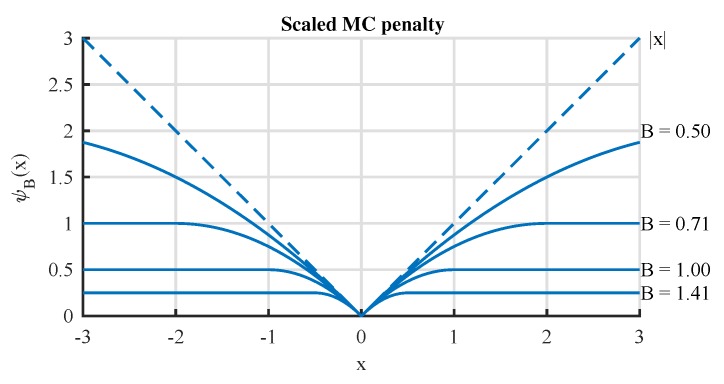
Illustration of the generalized minimax concave (GMC) penalty function.

Figure 2 illustrates the scalar version of GMC penalty functions. It shows that the GMC penalty has a basic property that large values are penalized no less than small values, but the degree of punishment is not as severe as $\ell_{1}$-norm [35]. Because of this property, it becomes possible to overcome the drawback of underestimating the high-amplitude components for the $\ell_{1}$-norm penalty. Moreover, the GMC penalty function is non-convex itself, but it can be carefully customized to maintain the convexity of the cost function to be minimized. This means that the global optimization of the cost function can be achieved with GMC penalty.

### 4.2. Problem Formulation

By replacing the $\ell_{1}$-norm penalty in Equation (1) with the GMC penalty Equation (9), we present the following sparsity recovery problem

(10) $$\arg\min_{x}\{\frac{1}{2}∥d_{w}-Ax{∥}_{2}^{2}+\lambda \psi_{B}\left(x\right)\}\;.$$

In order to maintain the convexity of the objective function, the parameters in *B* should be set carefully based on the following lemma.

**Lemma** **1.** *In [35], Let $y\in R^{M}$, $A\in R^{M\times N}$ and λ>0, the function $F\left(x\right)=\frac{1}{2}{∥y-Ax∥}_{2}^{2}+\lambda \psi_{B}\left(x\right)$ is a convex function if $B^{T}B⪯\frac{1}{\lambda }A^{T}A$. ($B^{T}B⪯\frac{1}{\lambda }A^{T}A$ implies that $A^{T}A-\lambda B^{T}B$ is positive semidefinite.)*

Based on Lemma 1 and given *A*, the convexity of the objective in Equation (10) can be satisfied by simply setting

(11) $$\begin{array}{c} B=\sqrt{\gamma /\lambda }A,0\leq \gamma \leq 1\;. \end{array}$$

- The non-convexity of the penalty $\psi_{B}$ is controlled by the parameter γ. When γ=0, the elements of *B* are zeros, and the penalty degenerates to the $\ell_{1}$-norm. When γ>0, a higher γ will lead to higher non-convexity.

### 4.3. Proposed GMC-Based Sparsity Recovery Algorithm

Similar with the classical $\ell_{1}$-norm penalty problem, there exists no closed-form solution for the GMC penalty problem Equation (10). In fact, the gradient of the GMC penalty is also inexplicit. In order to solve it, we substitute Equation (9) into Equation (10) and obtain a reformulated optimization problem

(12) $$\left(x^{opt},v^{opt}\right)=\arg\min_{x}\max_{v}F\left(x,v\right)$$

where $F\left(x,v\right)=\frac{1}{2}∥d_{w}{-Ax∥}_{2}^{2}+{\lambda ∥x∥}_{1}-{\lambda ∥v∥}_{1}-\frac{\gamma }{2}{∥A\left(x-v\right)∥}_{2}^{2}$ with 0≤γ≤1, and $d_{w}=\tilde{d}+\tilde{w}$.

Problem Equation (12) is a saddle-point optimization problem [44], which can also be solved by the FBS method [35,45]. We list details of the solving process in Algorithm 2.

**Figure 3:**
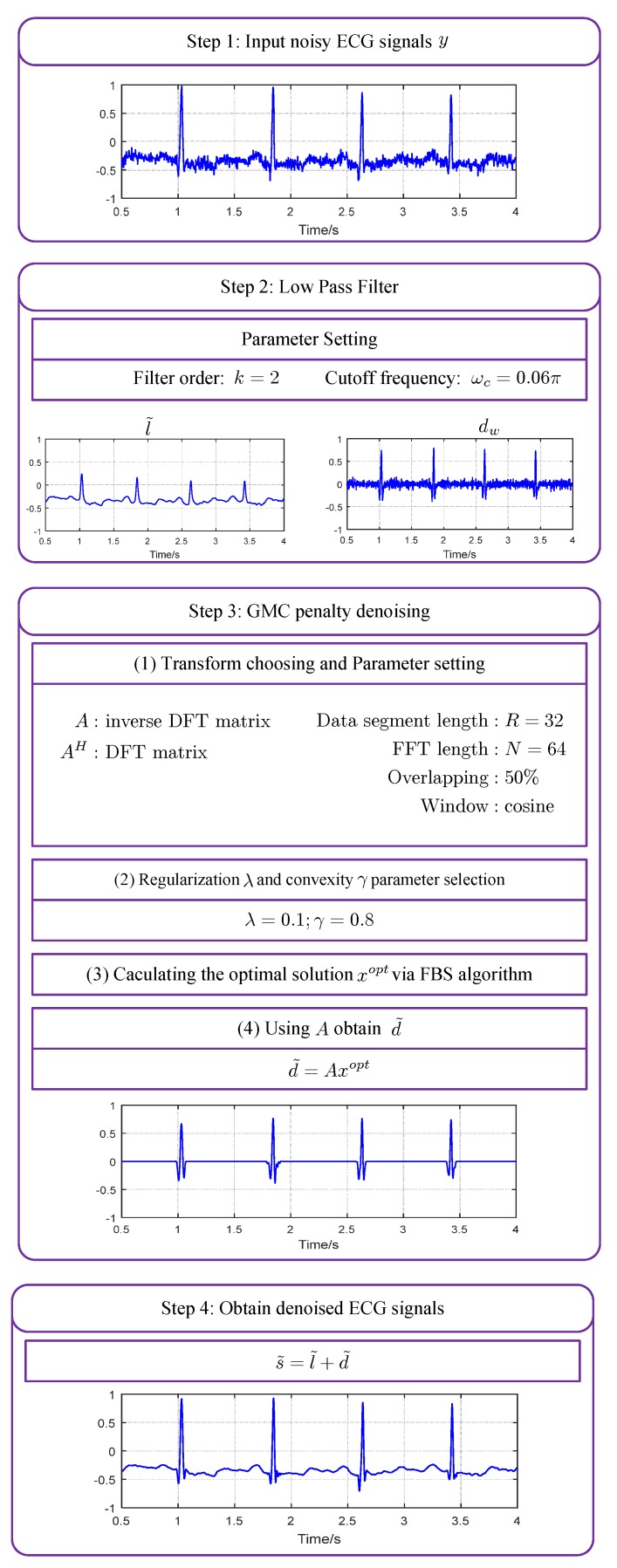
The detail flowchart of sparse ECG denoising with GMC-penalty.

Based on the sparse denoising framework in Figure 1 and the sparsity recovery algorithm in Algorithm 2, we propose the GMC-penalty ECG denoising method. The detailed processing flow of the method is shown in Figure 3 with some key parameter settings. The whole process consists of four steps. At the first step, the noisy ECG signal is sent to a low-pass filter. At the second step, the output of the LPF $\tilde{l}$ and the residual signal $y-\tilde{l}$ are obtained. Two parameters of the LPF, i.e., the order *k* and the cutoff frequency is $w_{c}$ should be configured beforehand. The next step is to perform sparse recovery following Algorithm 2. The linear transform matrix *A*, the data length *R*, the data overlapping ration and the window function should be set before applying the algorithm. Moreover, the regularization parameter λ and the convexity-guaranteeing factor γ are also set at the start of the algorithm. After processing $d_{w}$ by Algorithm 2, the optimal solution $x^{opt}$ is obtained for each data frame. Since $x^{opt}$ is the sparse coefficients in frequency domain, we should further recover the denoised sparse signal through the calculation $\tilde{d}=A^{H}x^{opt}$. Finally, we obtain the denoised ECG signal by synthesizing the low-pass component $\tilde{l}$ and the high-frequency sparse component $\tilde{d}$ in the last step.

**Algorithm 2** FBS algorithm for GMC penalized least squares problem**Input:** $x^{\left(0\right)},v^{\left(0\right)},A,A^{T},\lambda$**Output:** $x^{\left(i+1\right)}$
1:**Set** $\rho =max\left\{1,\gamma /\left(1-\gamma \right)\right\}\cdot maxeig\left(A^{T}A\right)$2:**Set** μ:0<μ<2/ρ3:Number of iteration: $N_{iter}$4:**for** $i=0\;to\;N_{iter}$**do**5: $w^{\left(i\right)}=x^{\left(i\right)}-\mu A^{T}\left(Ax^{\left(i\right)}-d_{w}\right)+\mu \gamma A^{T}A\left(v^{\left(i\right)}-x^{\left(i\right)}\right)$
6: $u^{\left(i\right)}=v\left(i\right)-\mu \gamma A^{T}A\left(v^{\left(i\right)}-x^{\left(i\right)}\right)$
7: $x^{\left(i+1\right)}=soft\left(w^{\left(i\right)},\mu \lambda \right)$
8: $v^{\left(i+1\right)}=soft\left(u^{\left(i\right)},\mu \lambda \right)$
9:
**end for**
10:
**return** $x^{\left(i+1\right)}$




## 5. Simulation Results

### 5.1. Configurations and Preprocessing

**Figure 4:**
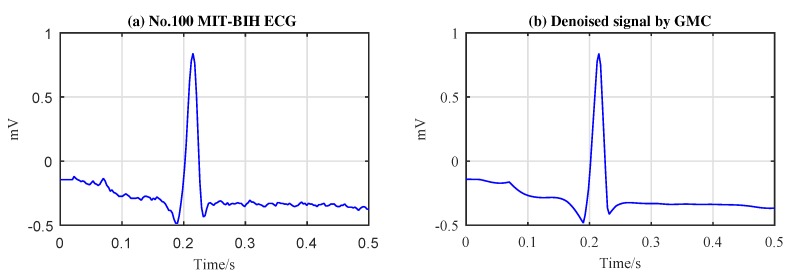
Denoising effect of original signal of MIT-BIH database.

The performance of the proposed algorithms are evaluated based on the ECG records of the MIT-BIH arrhythmia database [46]. The database contains a total of 48 records, each record length is 30 min, the sampling rate is 360 Hz. We note that the original signals in the MIT-BIH database have been filtered by a band-pass filter with a passband from 0.1 to 100 Hz. However, it is obvious that the noise components falling in the passband will not be eliminated. To understand its influence on the clean signal, we make an observation of the original signals in the database, as shown in Figure 4a. It can be observed that there exist noise components in the signals, but the amplitudes of the noise components are relatively weak compared with the useful signal. In order to gain deeper insight into the influence of the noise on our algorithms, we process the original signal directly using our algorithms, and the results are shown in Figure 4b. It can be perceived that the weak noise in the original signal is ‘smoothed’ out by our algorithms. That is to say our algorithms have the ability to suppress the noise in the acquired signals. However, considering that the original noise in the original signals of the database is relatively weak, we omit its impact on the ‘true’ signal. In this case, we call the original signals in the MIT-BIH database as ‘pure signal’, despite there existing very weak noise in practice.

The performance of the proposed algorithm is compared with the works in [30,47,48]. Three different performance criteria are chosen for the performance measurement, i.e., the rooted mean square error (RMSE), the percent root mean square difference (PRD) an the signal-to-noise ratio improvement $\left(SNR_{imp}\right)$.

For the LPF, we set the parameter of filter order as k=2. The the digital cutoff frequency is set as $w_{c}=0.06\pi$, i.e., $f_{c}=0.03*F_{s}=10.8$ Hz, where $F_{s}=360$ Hz is the sampling frequency of the ECG signals. It is noted that the cutoff frequency should be customized for different ECG signals, since the periodic characteristics of the ECG signals are not uniform in practice. We took data from the first 1 min, a total of 21,600 points. The data segment length *R* is set as 32, and the frame length of the STFT is 64.

**Figure 5:**
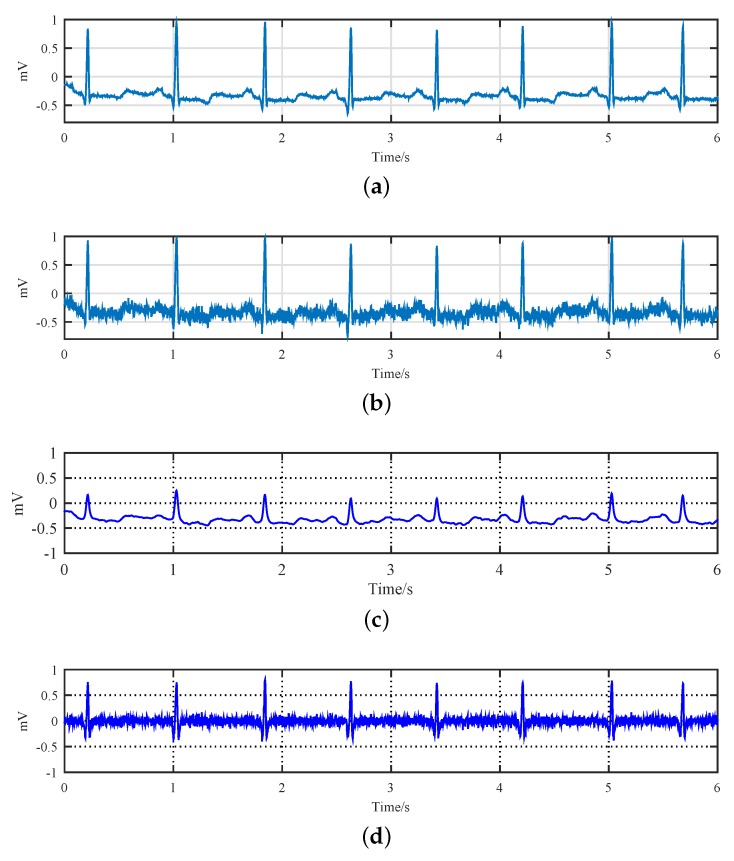
Comparison of the original ECG signal *y*, the noisy ECG signal *s*, the low-pass filtered signal $\tilde{l}$ and the residual sparse component $d_{w}$ in the proposed sparse denoising framework. (**a**) No.100 MIT-BIH ECG; (**b**) Noisy ECG with 10 dB SNR; (**c**) Output of the LPF; (**d**) Residual noisy sparse component.

To illustrate the proposed denoising framework, as shown by Figure 1, we plot the outputs of different preprocessing nodes in Figure 5. Specifically, Figure 5a is the original ECG signal read from the No.100 recode in the MIT-BIH database. This pure signal is then corrupted by AWGN noise with 10dB SNR and the result waveform is shown in Figure 5b. The noisy ECG signal, i.e., *s*, is the input of our denoising framework. It is sent to the LPF, and the output of which is shown in Figure 5c. By substituting this LPF signal from the raw noisy signal, a residual signal is obtained which can be treated approximately as a residual high-frequency sparse component of the ECG signal contaminated with noise, as shown in Figure 5d.

### 5.2. Convergence of the Proposed Sparsity Recovery Algorithms

**Figure 6:**
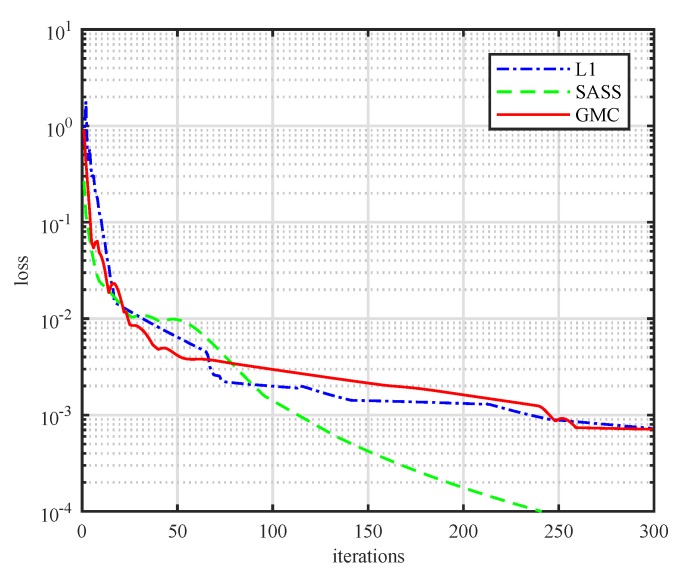
The updating rate of the recovered signal versus iterating numbers.

The noisy sparse signal $d_{w}=\tilde{d}+\tilde{w}$ is further sent to the sparsity recovery block in Figure 1, where the proposed $\ell_{1}$-norm method or the GMC-penalty method is applied to extract the sparse signal. The convergence curves of the algorithms are plotted in Figure 6. In the simulation, we set γ=0.8 and λ=0.1, and we define the update rate as $\frac{\tilde{d}\left(k\right)-\tilde{d}\left(k-1\right)}{\tilde{d}\left(k\right)}$, where *k* denotes the *k*th iteration. It can be observed that the updating rate of the algorithm will be less than 0.1% after about 250 iterations. This means the proposed algorithm will converge after hundreds of iterations. In Figure 6, we also plot the converge performance of the SASS method which is proposed in [30]. It can be observed that the update rate of the SASS algorithm will be less than $10^{-4}$ after 250 steps, which reveals that the proposed algorithms in this work converge slower than the SASS algorithm.

### 5.3. Performance Comparison of the Proposed Algorithms

**Figure 7:**
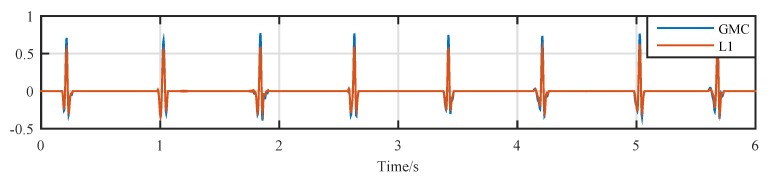
Output of the sparsity recovery block.

The outputs of the sparsity recovery block are plotted in Figure 7. It can be observed that the noise in the $d_{w}$ (i.e., Figure 5d is removed by both algorithms).

**Figure 8:**
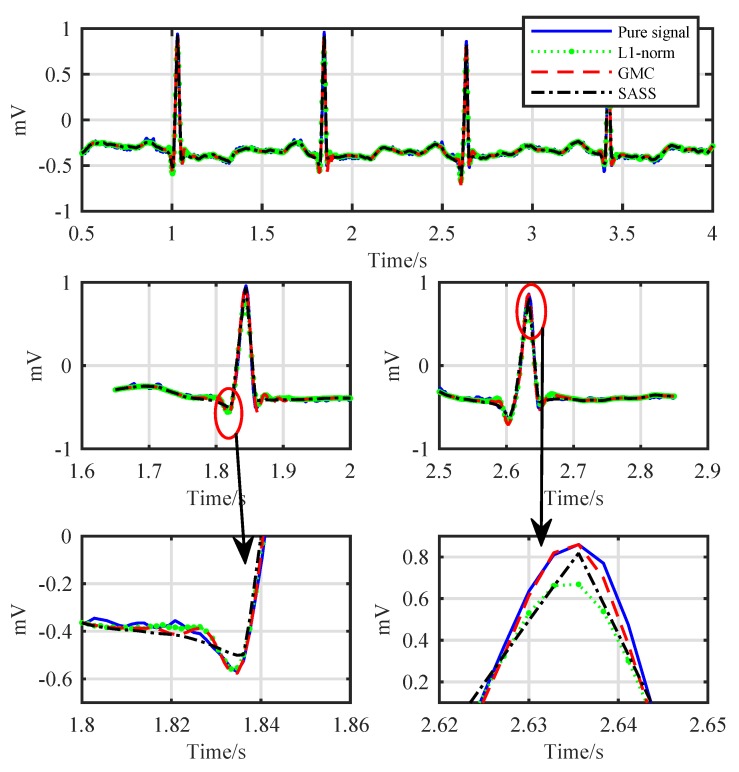
Comparison of the recovered signals in time domain.

As the final step of the proposed denoising scheme, we obtain the final denoised ECG signal by adding the outputs of the LPF and the sparsity recovery block according to Figure 1. The resulting waveforms are shown in Figure 8. It can be observed that all the three methods, i.e., the proposed $\ell_{1}$-norm method, the proposed GMC method and the SASS method, eliminate the noise effectively. However, the $\ell_{1}$-norm method underestimates the R-peaks visibly as shown in the down-right corner of Figure 8. The SASS method performs better than the $\ell_{1}$-norm method with respect to the R-peaks, but it underestimates the Q-peaks as shown in the down-left corner of Figure 8. As a comparison, the GMC method outperforms the other two methods with respect to both the R-peaks and Q-peaks.

**Figure 9:**
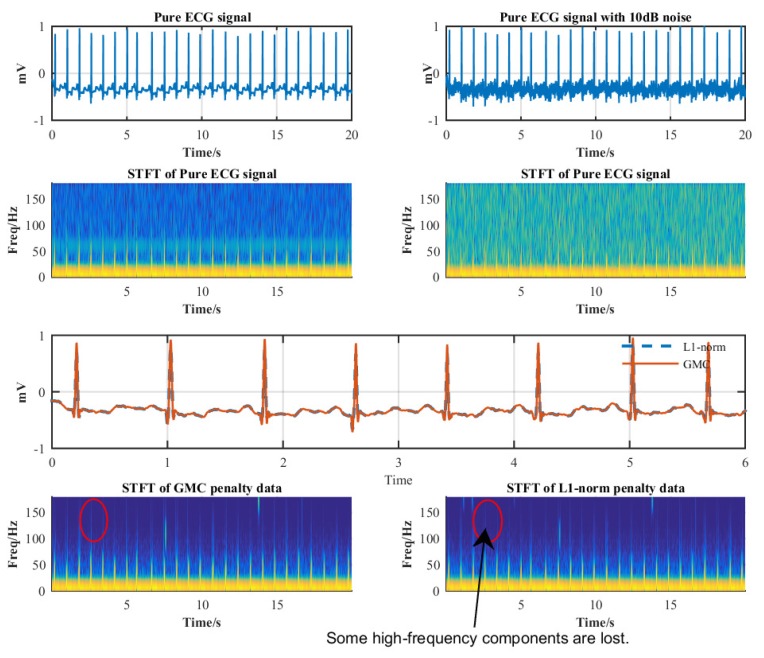
Comparison time-frequency properties of the GMC and $\ell_{1}$-norm methods.

To gain more insights into the proposed algorithms, we plot the time-frequency relationships of the recovered ECG signals in Figure 9. It is observed that the GMC-based method preserves more high-frequency components than the $\ell_{1}$-norm method, which can be perceived in the spectrogram.

### 5.4. Impacting of λ

**Figure 10:**
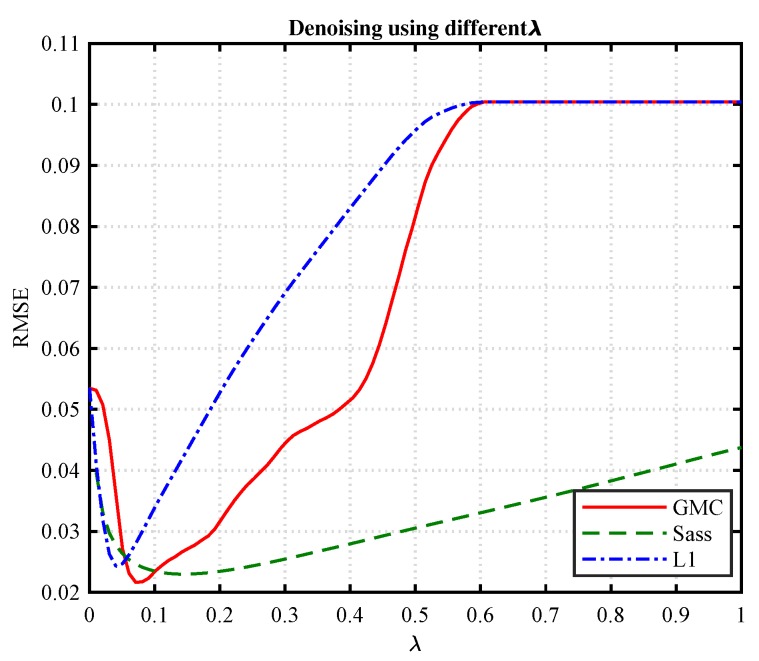
RMSE versus λ with γ=0.8.

For the GMC-based algorithm, both λ and γ should be specified. Since the two parameters are coupled, we can fix one of them and vary the other to study their impact on the performance of the method. Specifically, we set γ=0.8, and varying λ from 0 to 0.6. The resulted RMSE performance of the algorithm is shown in Figure 10. It can be observed that a better RMSE can be achieved when λ is chosen in the vicinity of 0.09.

**Figure 11:**
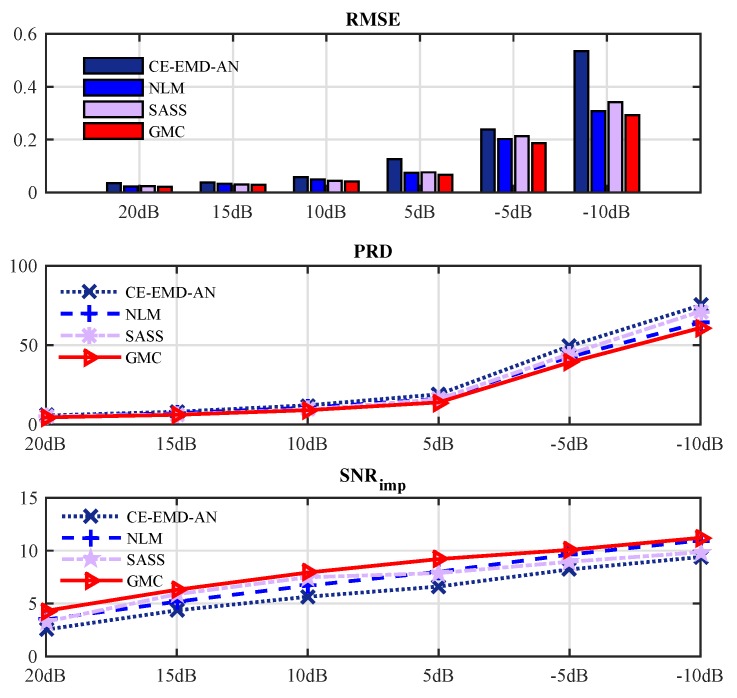
Performance evaluation over RMSE, PRD and $SNR_{imp}$ criteria.

In Figure 11, we compare the rooted mean square error (RMSE), the percent root mean square difference (PRD) and the signal-to-noise ratio improvement $\left(SNR_{imp}\right)$ achieved by different methods over different SNRs (20 dB 15 dB 10 dB 5 dB −5 dB −10 dB). The SASS [30], NLM [47] and CE-EMD-AN [48] algorithms are adopted as references. At each SNR, we average the results of all 48 data in the MIT-BIH database. It can be observed that the proposed GMC-based algorithm outperforms the referenced algorithms for all SNRs.

To fully demonstrate the performance of the proposed algorithm, we list the $SNR_{imp}$ values achieved by different algorithms over SNR = 10 dB in Table 1. The results show that the GMC-based algorithm performs the best of all of the experimented SNRs.

**Figure 12:**
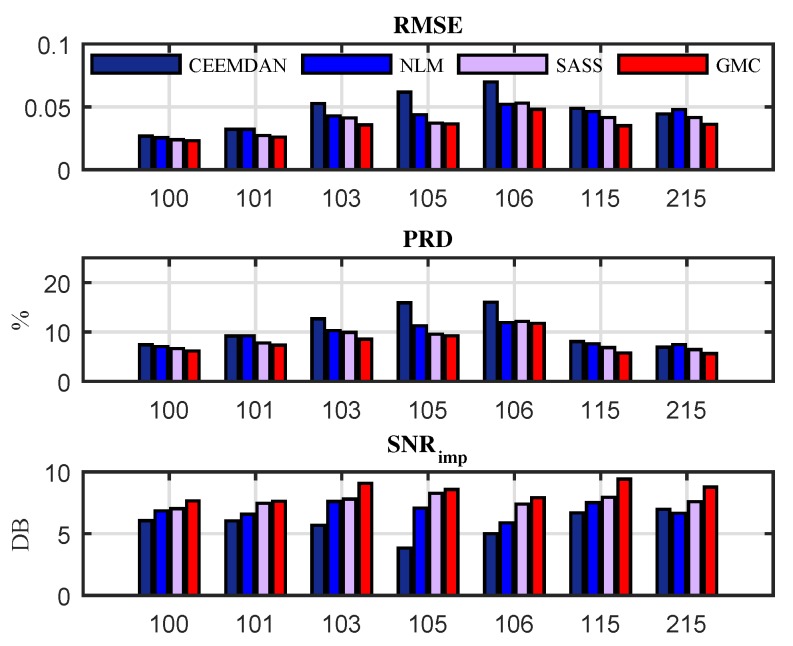
Performance evaluation over RMSE, PRD and $SNR_{imp}$ criteria for different data sources.

In order to more intuitively show the denoising performance of each algorithm, we illustrate in Figure 12 the values of RMSE, PRD and $SNR_{imp}$ achieved by the algorithms for the data number as 100, 101, 103, 105, 106, 115 and 220 in the database. It is noted that these data are selected because they were used in literature [47]. In fact, our algorithm can be applied to any ECG data in the database.

To further show performance of the the proposed GMC-based method, we compared the $SNR_{imp}$ percentage improvement of the GMC algorithm with the three referenced algorithms, i.e., SASS, NLM and CE-EMD-AN, under different noise levels. The percentage improvement is calculated according to the following formula We designed an experiment to calculate the percentage of GMC increase in $SNR_{imp}$ value relative to other algorithms. The calculation formula is as follows:

(13) $$\begin{array}{c} \mathrm{Percentage}\;\mathrm{improvement}=\frac{\mathrm{SNR}\_\mathrm{imp}\;\mathrm{of}\;\mathrm{GMC}-\mathrm{SNR}\_\mathrm{imp}\;\mathrm{of}\;\mathrm{the}\;\mathrm{referenced}\;\mathrm{algorithm}}{\mathrm{SNR}\_\mathrm{imp}\;\mathrm{of}\;\mathrm{the}\;\mathrm{referenced}\;\mathrm{algorithm}}\times 100\%. \end{array}$$

The results are listed in Table 2. It shows that the performance of the proposed algorithm outperforms the referenced algorithms significantly.

**Figure 13:**
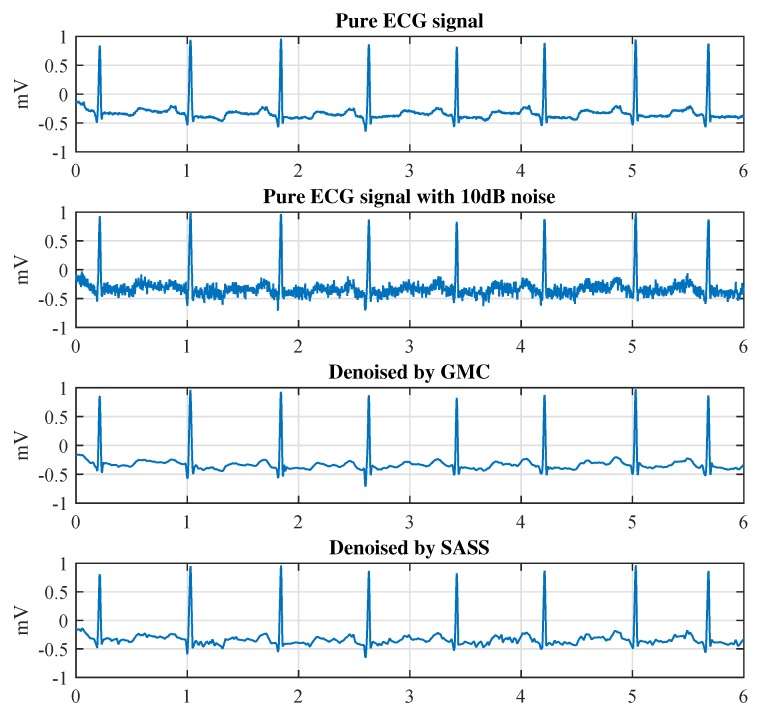

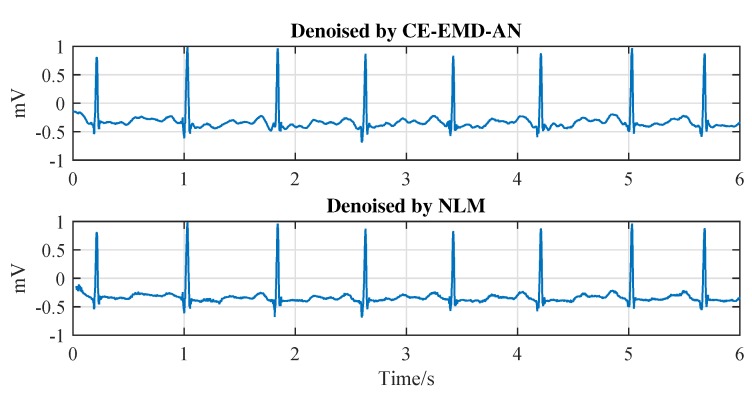
Comparison of waveform outputs of different algorithms for a normal ECG signal (No.100 in the MIT-BIH database).

**Figure 14:**
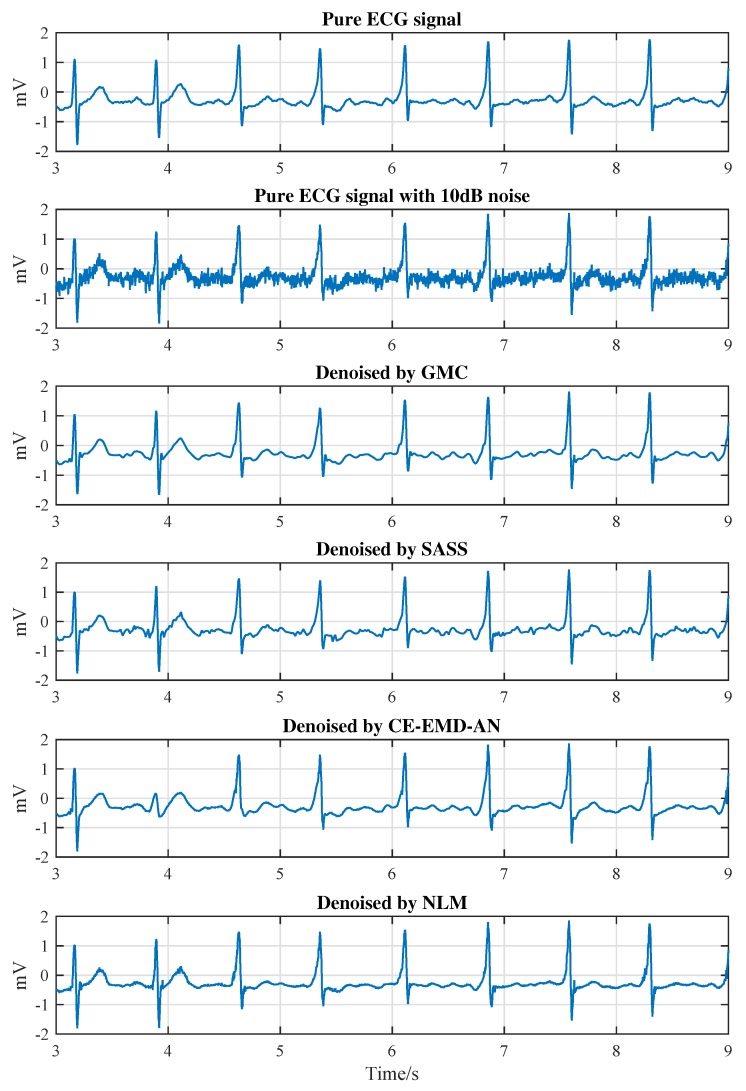
Comparison of waveform outputs of different algorithms for an arrhythmia ECG signal (No.230 in the MIT-BIH database).

There is a question that if the proposed algorithm can be applied to the abnormal ECG signals, such as arrhythmia signal, and how it will perform. To answer this question, we apply the proposed algorithm to the normal heart beat signal and the arrhythmia signal and results are shown in Figure 13 and Figure 14, respectively. As can be seen from Figure, all the four algorithms can remove noise effectively for the normal ECG signal. However, there is a huge difference in the type of signal that has an abnormal heart beat. As shown in Figure 14, after four seconds from the ECG signal (mitdb/230), the waveform begins to have turbulence and the T wave disappears significantly. After denoising, the output of the SASS algorithm is not smooth enough; the output of the CE-EMD-AN algorithm is seriously distorted in the period of 4 s–5 s; the output of the NLM algorithm has obvious sharp noise in the S-T segment during the 3 s–4 s period. Compared with these referenced algorithms, the proposed GMC algorithm achieves the best waveform. Therefore, the proposed GMC-based denoising algorithm can also be used with the arrhythmia signals.

## 6. Conclusions

We have investigated the ECG noise reduction techniques based on sparsity recovery in this paper. Through combining low-pass filtering and sparsity recovery, we proposed a novel sparse ECG denoising framework. The $\ell_{1}$ penalty and the GMC penalty were utilized to construct sparsity recovery algorithms. Although the GMC penalty function was non-differentiable non-convex itself, it could be used to construct convex cost function and thus helps to obtain optimal solution. Since the GMC had the property of penalizing large values more than small values, but the degree of punishment is not as severe as $\ell_{1}$-norm, it was utilized to overcome the drawback of underestimating the high-amplitude components for the $\ell_{1}$-norm penalty. Experimental results demonstrated that the proposed GMC-based method overcame the underestimating problem of the $\ell_{1}$-norm method and classical ECG denoising methods proposed in literature. Results showed that the GMC-based method outperforms the conventional algorithms such as the SASS algorithm and the EMD based method with respect to the $SNR_{imp}$, RMSE and PRD.

## Figures and Tables

**Table 1:** Comparison of the $SNR_{imp}$ achieved by different methods with input SNR = 10 dB.

Dateset	GMC	SASS	NLM	CEEMDAN	Dateset	GMC	SASS	NLM	CEEMDAN
**mitdb/100**	7.656	7.015	6.843	6.046	**mitdb/201**	8.212	7.505	6.619	6.361
**mitdb/101**	7.616	7.458	6.567	6.029	**mitdb/202**	8.530	8.121	7.704	7.596
**mitdb/102**	6.745	4.713	5.949	5.341	**mitdb/203**	5.805	5.336	4.537	3.448
**mitdb/103**	9.083	7.800	7.597	5.675	**mitdb/205**	8.105	7.196	6.236	5.088
**mitdb/104**	7.079	6.375	6.559	5.776	**mitdb/207**	9.252	8.541	8.224	6.721
**mitdb/105**	8.576	8.267	7.073	3.828	**mitdb/208**	7.838	8.382	6.324	5.050
**mitdb/106**	7.912	7.382	5.867	4.992	**mitdb/209**	7.632	7.276	6.085	4.457
**mitdb/107**	7.925	8.188	7.450	6.923	**mitdb/210**	8.183	8.083	7.170	4.965
**mitdb/108**	6.962	6.921	6.297	4.352	**mitdb/212**	7.166	7.042	5.938	4.612
**mitdb/109**	8.326	8.394	7.565	7.559	**mitdb/213**	8.217	8.158	7.134	4.872
**mitdb/111**	7.162	6.761	6.200	5.476	**mitdb/214**	8.410	8.292	7.462	4.706
**mitdb/112**	8.318	7.826	6.682	5.680	**mitdb/215**	7.168	7.181	6.116	5.443
**mitdb/113**	8.668	8.098	7.353	5.224	**mitbd/217**	7.652	7.688	6.116	5.645
**mitdb/114**	6.102	6.032	5.371	4.885	**mitdb/219**	8.667	7.963	7.723	4.377
**mitdb/115**	9.417	7.948	7.503	6.675	**mitdb/220**	8.767	7.586	6.653	6.977
**mitdb/116**	7.825	7.434	6.823	3.651	**mitdb/221**	8.916	7.632	6.670	5.163
**mitdb/117**	8.122	8.136	6.506	7.973	**mitdb/222**	6.596	6.566	5.909	5.200
**mitdb/118**	7.366	5.043	6.078	5.774	**mitdb/223**	8.580	7.920	7.540	7.163
**mitdb/119**	8.575	7.842	7.504	6.274	**mitdb/228**	6.777	7.549	6.752	6.039
**mitdb/121**	9.702	8.525	7.598	4.631	**mitdb/230**	8.209	7.860	6.376	5.844
**mitdb/122**	7.856	7.401	7.048	7.105	**mitdb/231**	8.076	7.859	7.225	6.152
**mitdb/123**	9.007	7.692	7.037	6.093	**mitdb/232**	2.206	4.824	3.741	3.666
**mitdb/124**	9.252	8.164	7.374	6.858	**mitdb/233**	8.973	8.894	6.500	6.305
**mitdb/200**	8.190	8.134	6.638	5.842	**mitdb/234**	8.265	8.193	7.014	6.284

**Table 2:** Percentage $SNR_{imp}$ improvement relative to the three referenced algorithms.

	20 dB	15 dB	10 dB	5 dB	−5 dB	−10 dB
**SASS**	21.32	6.60	5.12	13.94	10.68	11.89
**NLM**	21.59	17.86	15.38	13.04	4.37	2.36
**CE-EMD-AN**	42.50	30.94	28.67	28.23	18.28	16.37

## Abbreviations

The following abbreviations are used in this manuscript:

GMC	Generalized Minimax Concave Penalty
GSNC	Grey Spectral Noise Cancellation
GSNE	Grey Spectral Noise Estimation
ECG	Electrocardiogram
EMD	Empirical Mode Decomposition
SASS	Sparsity Assisted Signal Smoothing
NLM	Non-local Means
IMFs	Intrinsic Mode Functions
LTI	Linear Time Invariant
AWGN	Additive White Gaussian Noise
LPF	Low-pass Filter
HPF	High-pass Filter
STFT	Short Time Fourier Transform
BPD	Basis Pursuit Denoising
FBS	Forward-backward Spliting
SNR	signal-to-noise ratio
$SNR_{imp}$	signal-to-noise ratio improvement
RMSE	average of root mean square error
PRD	Percent root mean square difference

## Appendix A. Filter Designing

In this paper, we take the LPFs to be zero-phase, non-causal, recursive filters considering that they filter relatively symmetric signals without introducing shifts in peak locations [29,38,49]. Recently, a novel procedure for defining such filters is given in [30]. We briefly sketch its key design idea here.

Given finite-length sequences $p_{n}$ and $q_{n}$, two banded Toeplitz matrices *P* and *Q* are defined as

(A1) $$P=\left[\begin{array}{cccccc} p_{1} & p_{2} & p_{3} & & & \\ & p_{1} & p_{2} & p_{3} & & \\ & & & \ddots & & \\ & & & p_{1} & p_{2} & p_{3} \end{array}\right]$$

(A2) $$Q=\left[\begin{array}{cccccc} q_{1} & q_{2} & q_{3} & & & \\ & q_{1} & q_{2} & q_{3} & & \\ & & & \ddots & & \\ & & & q_{1} & q_{2} & q_{3} \end{array}\right]\;.$$

Based on these matrices, a linear filter matrix can be constructed as

(A3) $$\begin{array}{c} H={\left(Q^{T}Q+\alpha P^{T}P\right)}^{-1}Q^{T}Q\;. \end{array}$$

The matrix *H* is approximately Toeplitz, and it represents approximately an LTI system with the frequency response

(A4) $$\begin{array}{c} H\left(e^{j\omega }\right)=\frac{|Q\left(e^{j\omega }\right){|}^{2}}{|Q\left(e^{j\omega }\right){|}^{2}+\alpha {\left|P\left(e^{j\omega }\right)\right|}^{2}}\;. \end{array}$$

$H(e^{j\omega })$ is zero-phase, and its transfer function is written as

(A5) $$\begin{array}{c} H\left(z\right)=\frac{Q\left(z\right)Q\left(z^{-1}\right)}{Q\left(z\right)Q\left(z^{-1}\right)+\alpha P\left(z\right)P\left(z^{-1}\right)}\;. \end{array}$$

After implementing Equation (A3) to the input data vector *y* (e.g., the noisy data), the vectorized output of the filter is written as

(A6) $$\begin{array}{c} x=Hy\;. \end{array}$$

An advantage of Equation (A6) is that transient effects at the start and end of the finite-length signal are intrinsically avoided. This means that the transient end-point artifacts is avoided.

In order to construct the transfer functions of the form Equation (A5), P(z) and Q(z) can be set as

(A7) $$\begin{array}{c} P\left(z\right)={\left(1-z^{-1}\right)}^{k}\;, \end{array}$$

(A8) $$\begin{array}{c} Q\left(z\right)={\left(1+z^{-1}\right)}^{k}\;, \end{array}$$

where *k* is the order of the filter. It is easy to verify that P(1)=0 and Q(−1)=0, H(1)=1 and H(−1)=0. Hence, the frequency response of the filter *H* has unity gain at ω=0 and zero at ω=π.

Given Equations (A7) and (A8), the filter *H* is a discrete-time Butterworth filter with the frequency response

(A9) $$\begin{array}{c} H\left(e^{j\omega }\right)=\frac{\cos^{2k}\left(\omega /2\right)}{\cos^{2k}\left(\omega /2\right)+\alpha \sin^{2k}\left(\omega /2\right)}\;, \end{array}$$

where α controls the cut-off frequency. For the 3 dB cutoff frequency, i.e., $H(e^{j\omega_{c}})=0.5$, α can be set as

(A10) $$\begin{array}{c} \alpha =1/\tan^{2k}\left(w_{c}/2\right)\;. \end{array}$$

## Appendix B. The STFT Used in the Sparsity Recovery

We use the STFT in the sparsity recovery block in Figure 1. Specifically, the input of the sparsity recovery block is cut as small data segments with length *R*. The segments are overlapped with a ratio of 50%. After padding *R* zeros behind the tail of the data segment, a STFT frame with length N=2R is constructed.

The constructed time-domain signal frame $d\in R^{N}$ can be represented by the frequency-domain signal $x\in C^{N}$ as

(A11) $$\begin{array}{c} d=Ax\;, \end{array}$$

where $A\in C^{N\times N}$ is the inverse discrete Fourier transform (IDFT) matrix defined as

(A12) $$\begin{array}{c} A=\frac{1}{\sqrt{N}}{\left[e^{j\frac{2\pi }{N}kn}\right]}_{k=0,\ldots ,N-1;n=0,\ldots ,N-1}\;, \end{array}$$

The time-domain representation Equation (A11) can be transformed into the frequency-representation as

(A13) $$\begin{array}{c} x=A^{H}d\;. \end{array}$$

It is noted specially that a window function with length *R* is applied to the segmented time-domain data, which is defined as

(A14) $$\begin{array}{c} w\left(n\right)=\sin\left(\frac{n+0.5}{R}\pi \right),n=0,\ldots ,R-1\;. \end{array}$$

After the frequency-to-time transform, this window should be removed by multiplexing its reciprocal.
